# Identification of potential biomarkers for cerebral palsy and the development of prediction models

**DOI:** 10.3389/ebm.2024.10101

**Published:** 2024-07-09

**Authors:** Haoyang Zheng, Duo Zhang, Yong Gan, Zesheng Peng, Yuyi Wu, Wei Xiang

**Affiliations:** ^1^ Department of Neurosurgery, Union Hospital, Tongji Medical College, Huazhong University of Science and Technology, Wuhan, China; ^2^ Department of Nursing, Tongji Hospital, Tongji Medical College, Huazhong University of Science and Technology, Wuhan, Hubei, China; ^3^ Department of Social Medicine and Health Management, School of Public Health, Tongji Medical College, Huazhong University of Science and Technology, Wuhan, China

**Keywords:** cerebral palsy, neurodevelopmental disorder, biomarkers, prediction models, therapeutic targets

## Abstract

Cerebral palsy (CP) is a prevalent motor disorder originating from early brain injury or malformation, with significant variability in its clinical presentation and etiology. Early diagnosis and personalized therapeutic interventions are hindered by the lack of reliable biomarkers. This study aims to identify potential biomarkers for cerebral palsy and develop predictive models to enhance early diagnosis and prognosis. We conducted a comprehensive bioinformatics analysis of gene expression profiles in muscle samples from CP patients to identify candidate biomarkers. Six key genes (CKMT2, TNNT2, MYH4, MYH1, GOT1, and LPL) were validated in an independent cohort, and potential biological pathways and molecular networks involved in CP pathogenesis were analyzed. The importance of processes such as functional regulation, energy metabolism, and cell signaling pathways in the muscles of CP patients was emphasized. Predictive models of muscle sample biomarkers related to CP were developed and visualized. Calibration curves and receiver operating characteristic analysis demonstrated that the predictive models exhibit high sensitivity and specificity in distinguishing individuals at risk of CP. The identified biomarkers and developed prediction models offer significant potential for early diagnosis and personalized management of CP. Future research should focus on validating these biomarkers in larger cohorts and integrating them into clinical practice to improve outcomes for individuals with CP.

## Impact statement

The discovery of reliable biomarkers has the potential to revolutionize clinical practice by enabling earlier and more accurate diagnosis of CP, which can lead to timely and targeted therapeutic interventions. Early identification of at-risk individuals allows for the implementation of neuroprotective strategies and tailored rehabilitation programs, potentially mitigating the severity of motor impairments and improving long-term outcomes. This study’s findings set the stage for future research to validate and refine these biomarkers in larger, diverse populations. Ultimately, the integration of biomarker-based diagnostics into routine clinical practice could transform the management of cerebral palsy, offering new hope for improved quality of life for affected individuals and their families.

## Introduction

Cerebral palsy (CP) remains one of the most prevalent childhood motor disorders, affecting approximately 2–2.5 per 1,000 live births worldwide [1]. It encompasses a heterogeneous group of non-progressive disorders of movement and posture caused by early brain injury or malformation, with implications for motor function throughout an individual’s lifespan [2]. Despite extensive research, the etiology of CP often remains elusive, hindering both early diagnosis and the implementation of targeted therapeutic interventions.

Skeletal muscles in patients with CP are altered due to neurological lesions. These brain lesions cause various neurological symptoms, including dystonia, ataxia, athetosis, and particularly spasticity [3, 4]. Loss of upper motor neuron inhibition on the lower motor neurons resulted in spasticity, altered muscle tone, and increased or impaired motor unit firing [5]. Although the mechanism is unknown, spastic muscle often shortens to create muscle contractures, which is a primary disability of CP that leads to further complications. CP is the most prevalent non-genetic cause of secondary dystonia, and its clinical management poses significant challenges [6]. The primary objectives in treating dystonia associated with CP are to mitigate dystonic symptoms, optimize functional capacity, alleviate pain, and enhance overall care convenience [7]. Oral medications, physical therapy techniques, chemical neurectomies with phenol or alcohol, chemodenervation using neurotoxins, and deep brain stimulation have been utilized to decrease spasticity and dystonic symptoms among children with CP, but often yield suboptimal results [8].

Skeletal muscle in patients with CP exhibits distinct characteristics, including muscle tissue and fiber atrophy, decreased cross-sectional area, muscle shortening, and reduced specific tension [9]. Identifying reliable biomarkers associated with CP is crucial for understanding its diverse etiologies, facilitating early diagnosis, prognostication, and targeted therapeutic interventions. However, the identification of reliable biomarkers and their translation into clinical practice remain significant challenges.

This study aimed to address these challenges by systematically identifying potential biomarkers for CP and developing robust prediction models. By leveraging advanced computational algorithms, we sought to uncover biomarkers that could serve as reliable indicators of CP risk and severity. In this study, we provided a detailed description of our methods for the discovery of biomarkers and the development of predictive models. We discussed the implications of the findings for clinical practice and proposed strategies for the future integration of biomarker-based diagnostics in the management of CP.

## Materials and methods

### Data acquisition and preprocessing

**FIGURE 1:**
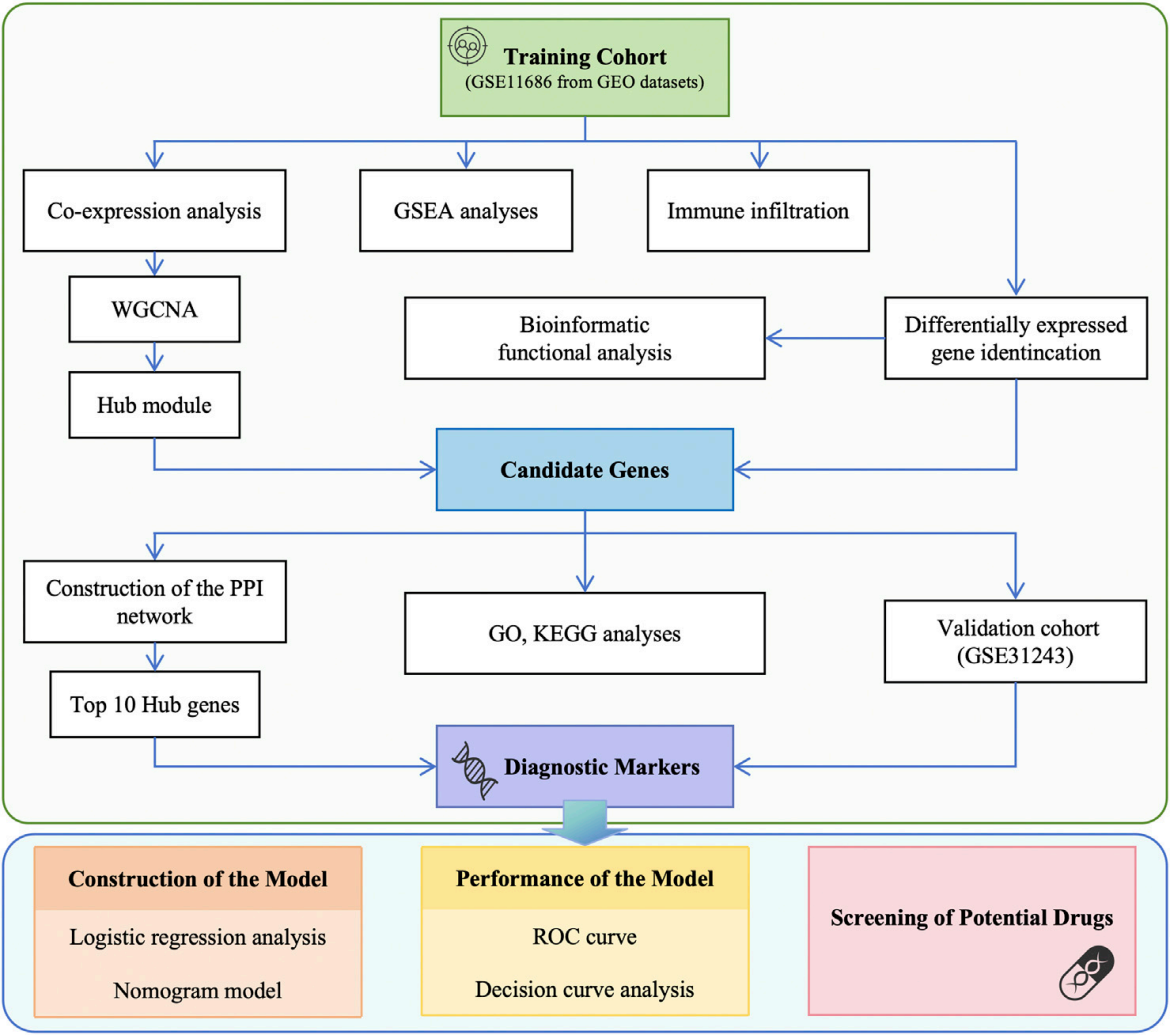
Flowchart of data preparation and analysis in this study. GEO, Gene Expression Omnibus; WGCNA, weighted gene co-expression network analysis; GSEA, gene set enrichment analysis; PPI, protein-protein interaction; GO, Gene ontology; KEGG, Kyoto Encyclopedia of Genes and Genomes; ROC, receiver operating characteristic; DCA, decision curve analysis.

The data used in this article was obtained from the NCBI Gene Expression Integration (GEO) database. The following criteria were used for screening the datasets: (1) inclusion of samples from CP patients and healthy individuals, (2) focus on muscle tissue gene expression profiles, (3) availability of publicly accessible raw or processed data, (4) research conducted on *Homo sapiens*, (5) total sample size greater than 15, and (6) exclusion of samples associated with other diseases. Two different gene expression datasets were analyzed in this study: GSE11686 [10] as the analysis set and GSE31243 [11] as the validation set. Detailed characteristics are shown in Table 1. To ensure an adequate sample size and the generalizability of the results, we included data from different muscle samples and performed quality control, preprocessing, and statistical analysis using the limma package in R Studio. The data analysis workflow is depicted in Figure 1.

**TABLE 1:** Detailed characteristics of the included data sets.

Sample ID	Cohort	Patients		Controls		Tissue of sample
		Sample size	Age (mean ± SD)	Sample size	Age (mean ± SD)	
GSE11686	Training	6	10.3 ± 3.79	2	8.5 ± 2.1	Wrist muscle extensors and flexors
GSE31243	Validation	10	14.8 ± 1.25	10	12.8 ± 1.5	Gracilis and semitendinosus

Note: SD, standard deviation.

### Identification of the differentially expressed genes (DEGs)

DEGs between the CP group and the control group were identified using the limma package and visualized with a volcano plot. Genes were selected for further analysis in the network construction based on the significance analysis of microarrays (SAM) with adjusted *p*-value < 0.05 and |log2 fold change (FC)| ≥ 1.2. A heatmap of the DEGs that were screened was generated in R software.

### Gene set enrichment analysis (GSEA)

To provide a clearer representation of the gene expression level of highly enriched functional pathways, we used the GSEA software (version 3.0) and downloaded the sub-aggregate of c2.cp.kegg.v7.4.symbols.gmt. from the Molecular Signatures Database (DOI:10.1093/bioinformatics/btr260^1^ [12]. The minimum gene set was 5, the maximum gene set was 5,000, and 1,000 resampling was performed. A *p*-value of <0.05 was considered statistically significant.

### Functional enrichment analysis

The DEGs were subjected to functional enrichment analysis using DAVID^2^. Gene ontology (GO) analysis was performed to identify distinguishing biological characteristics, including molecular functions (MF), biological pathways (BP), and cellular components (CC). Kyoto Encyclopedia of Genes and Genomes (KEGG) enrichment analysis was used to explore the activities of genes and their connections to high-level genomic information.

### Evaluation and correlation analysis of infiltration-related immune cells

The infiltration matrix of immune cells was obtained by filtering 22 types of immune cell matrices using the cell-type identification by estimating relative subsets of RNA transcripts (CIBERSORT) website (*p* < 0.05) [13]. The Spearman correlation analysis was conducted between unique diagnostic markers and immune infiltrating cells using the “ggplot2” package to illustrate the results.

### Construction of weighted gene co-expression network and identification of significant modules

The weighted gene co-expression network analysis (WGCNA) is a valuable tool for studying gene set expression. Data were processed using R-Studio 4.2.2, and abnormal samples were excluded for reliability. Samples were clustered to identify outliers, and the network was built using the automatic network construction function, which determined the soft threshold power β. Adjacency was calculated based on co-expression similarity. Hierarchical clustering created a tree diagram with modules, which were automatically merged for highly correlated feature genes (TOM type = “unsigned,” min module size = 30, merge cut height = 0.25). Genes with similar expression patterns were grouped into modules, each assigned a specific color. Module membership (MM) and gene significance (GS) were calculated for clinically relevant modules. Gene information from these modules was extracted for further analysis, and the characteristic gene network was visualized.

### Identification of candidate genes

The Venn diagram shows the intersection of WGCNA brown modular genes and DEGs, representing disease-related genes and differentially expressed genes. In total, 45 genes were identified as candidate genes, and their expression is shown in Table 2.

**TABLE 2:** The gene expression levels of 45 overlap hub genes.

Gene symbol	*p*-Value	Log FC	Gene title
ACYP1	0.028656	1.273,244	Acylphosphatase-1
ADM	0.017645	−1.225,876	Adrenomedullin
ALCAM	0.018667	1.224,642	Activated leukocyte cell adhesion molecule
AMOT	0.015123	−1.308,099	Angiomotin
ASTN2	0.031953	1.476,799	Astrotactin-2
BDH1	0.033195	−1.561,303	3-hydroxybutyrate dehydrogenase 1
CA8	0.005997	−1.429,715	Carbonic anhydrase VIII
CHAD	0.002794	2.096462	Chondroadherin
CKB	0.008580	−1.320,115	Creatine kinase B-type
CKMT2	0.001798	−1.218,961	Creatine kinase S-type, mitochondrial
CRYM	0.007519	−2.363,445	Crystallin, mu
ESPN	0.006668	−1.604,240	Espin
FABP3	0.009801	−1.800,821	Fatty acid binding protein 3
GOT1	0.013254	−1.301,036	Glutamic-oxaloacetic transaminase 1
GPX3	0.009929	−1.383,203	Glutathione peroxidase 3
HIST1H2BE	0.001751	1.247,343	Histone cluster 1, H2be
KAL1	0.000630	1.337,068	Kallmann syndrome 1 sequence
KCNN2	0.007283	1.591,455	Small conductance calcium-activated potassium channel protein 2
LDHB	0.003401	−1.226,687	Lactate dehydrogenase B
LPL	0.011452	−1.648,013	Lipoprotein lipase
MAP3K7CL	0.010553	1.571,485	MAP3K7 C-terminal like
MMRN1	0.016738	−1.624,806	Multimerin 1
MPC1	0.007283	−1.235,675	Mitochondrial pyruvate carrier 1
MYH1	0.037308	2.365,836	Myosin-1
MYH4	0.001802	2.046775	Myosin-4
NAP1L2	0.005890	1.887,987	Nucleosome assembly protein 1-like 2
NINJ1	0.001699	1.290,459	Ninjurin-1
NKAIN1	0.014994	1.677,495	Na+/K+ transporting ATPase interacting 1
NME3	0.034381	1.597,844	Nucleoside diphosphate kinase 3
NOTCH2NL	0.039355	1.217,319	Notch homolog 2 N-terminal-like protein A
NPTX2	0.019633	−1.319,154	Neuronal pentraxin-2
NSUN5P1	0.028064	1.232,678	Putative NOL1/NOP2/Sun domain family member 5B
OMD	0.008529	1.404,630	Osteomodulin
PEG10	0.006821	1.203,384	Paternally expressed 10
POLI	0.005709	1.571,104	Polymerase (DNA directed) iota
POLM	0.001989	−1.283,703	DNA-directed DNA/RNA polymerase mu
PPIF	0.004543	−1.211,272	Peptidyl-prolyl cis-trans isomerase F
PREB	0.009929	−1.241,452	Prolactin regulatory element-binding protein
PVALB	0.001802	4.740,443	Parvalbumin alpha
RETSAT	0.000630	−1.540,679	All-trans-retinol 13,14-reductase
SLC12A8	0.036487	−1.651,596	Solute carrier family 12, member 8
SP140L	0.002429	1.601,102	SP140 nuclear body protein like
TGM2	0.001802	−1.509,241	Protein-glutamine gamma-glutamyltransferase 2
TNNT2	0.026367	1.338,269	Troponin T type 2 (cardiac)
TST	0.001802	−1.352,077	Thiosulfate sulfurtransferase

### Protein-protein interaction (PPI) network construction and identification of hub genes

To identify the hub genes of each module, the previously acquired genes were mapped to the STRING database^3^, a platform for searching PPI. The protein interactions of each module were then constructed and visualized using the CytoHubba plugin within the Cytoscape software^4^. The hub gene was determined as the one with the highest degree of connection. In this study, the Maximal Clique Centrality (MCC) method in CytoHubba, known for its accuracy in predicting essential proteins, was used [14].

### Validation of the hub genes expression and prediction value

To validate the expression differences of the hub genes and their universality, we utilized gene expression data from GSE31243, which consists of 20 CP and 20 non-CP muscle samples. The expression of hub genes in muscle samples from CP and non-CP patients was analyzed using box plots created with the “ggplot2” package in R software. The data were presented as standard deviation. Statistical analysis was performed using an unpaired independent *t*-test, with a significance level set at *p* < 0.05.

### Establishment and validation of prediction models and nomogram

To establish the prediction model, we utilized logistic regression analysis. The multivariate model included hub genes that showed differential expression in both the training and validation cohorts. Based on the regression coefficients of the relevant genes in the training cohort, we developed a nomogram. Model covariates were assigned points in the range of 0–100, corresponding to their values. The total points obtained from the predictive model indicated the risk of CP. We assessed the performance of the nomogram using the calibration curve in the training cohort. The predictive ability of the model was evaluated in both the training and validation cohorts using the area under the ROC curve (AUC). We generated ROC curves using SPSS. Genes were considered to have potential clinical significance if their AUC was greater than 0.6.

### Prediction of potential drugs

Based on the biomarkers of CP, the DGIdb database^5^ was utilized to predict potential drugs for the treatment of CP. The network of biomarker-compound pairs was visualized using the Cytoscape software.

## Results

### GSEA

**FIGURE 2:**
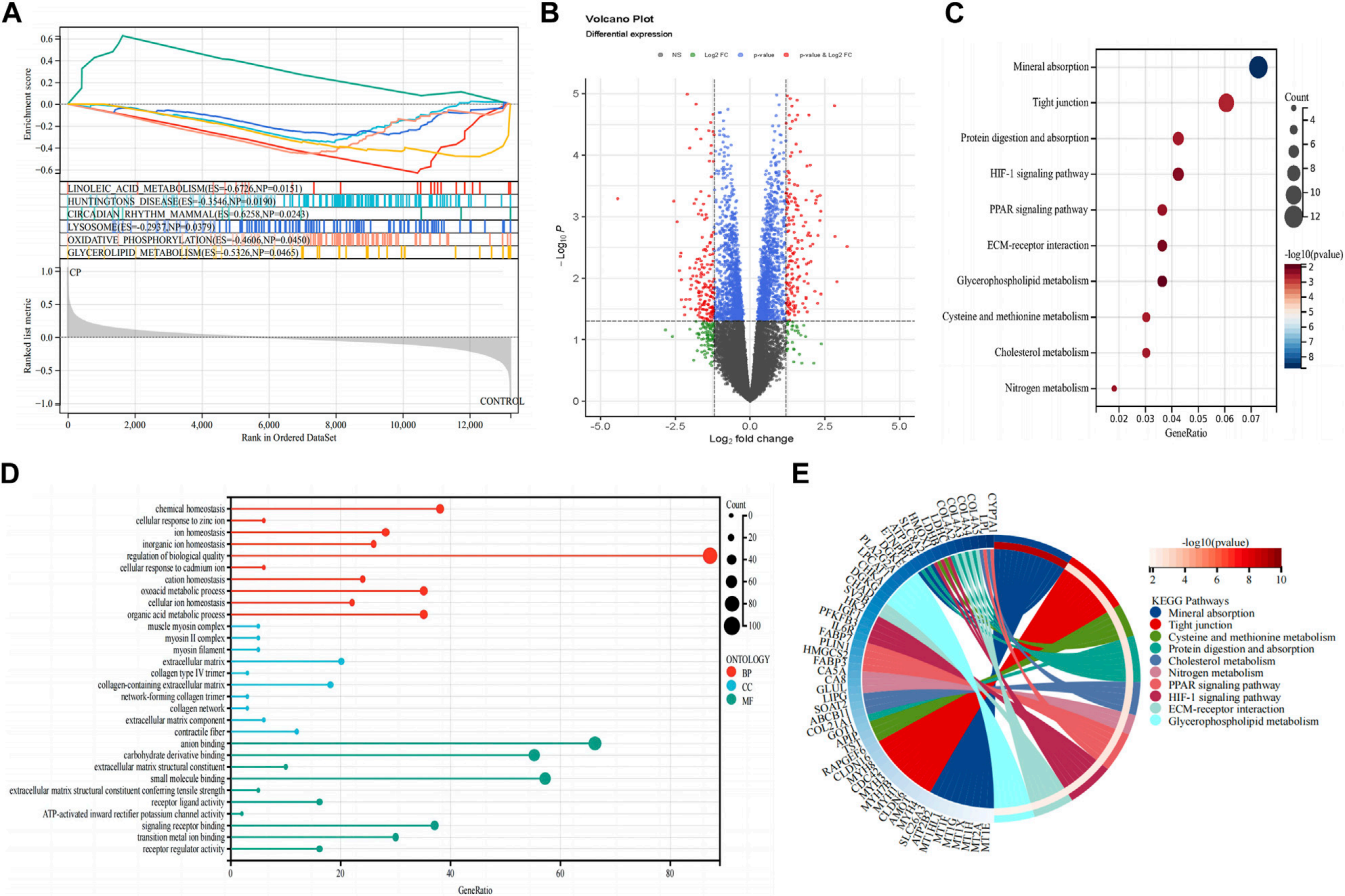
Detection of differentially expressed genes and functional enrichment analysis. **(A)** GSEA analysis; **(B)** Volcano plot of the 353 DEGs; **(C)** KEGG pathway enrichment analysis; **(D)** GO enrichment analysis; **(E)** The KEGG-enriched chord diagram shows the genes involved in the KEGG term. DEGs, differentially expressed genes; FC, fold-change; GSEA, gene set enrichment analysis; GO, Gene ontology; KEGG, Kyoto Encyclopedia of Genes and Genomes.

GSEA was conducted on both patients with CP and healthy control subjects to investigate the biological signaling pathway. The top five terms are shown in Figure 2A. Linoleic acid metabolism, Huntington’s disease, circadian rhythm, lysosome, oxidative phosphorylation, and glycerolipid metabolism were significantly enriched in the patients with CP.

### Functional enrichment analysis of DEGs

A total of 353 DEGs were identified, including 173 upregulated and 180 downregulated genes (Figure 2B). We performed functional analysis to gain a deeper understanding of the biological functions of the DEGs. In terms of BP, the clusters were significantly associated with the regulation of biological quality, chemical homeostasis, and organic acid metabolic process. In the MF analysis, our results indicate that the DEGs are significantly associated with anion binding, small molecule binding, and carbohydrate derivative binding. In the CC enrichment analysis, the focus was on the extracellular matrix (ECM), collagen-containing ECM, and contractile fiber (Figure 2D). In the KEGG pathway analysis (Figures 2C, E), mineral absorption, tight junction, and protein digestion and absorption were identified as significant pathways in the DEGs.

### Infiltration of immune cells results

**FIGURE 3:**
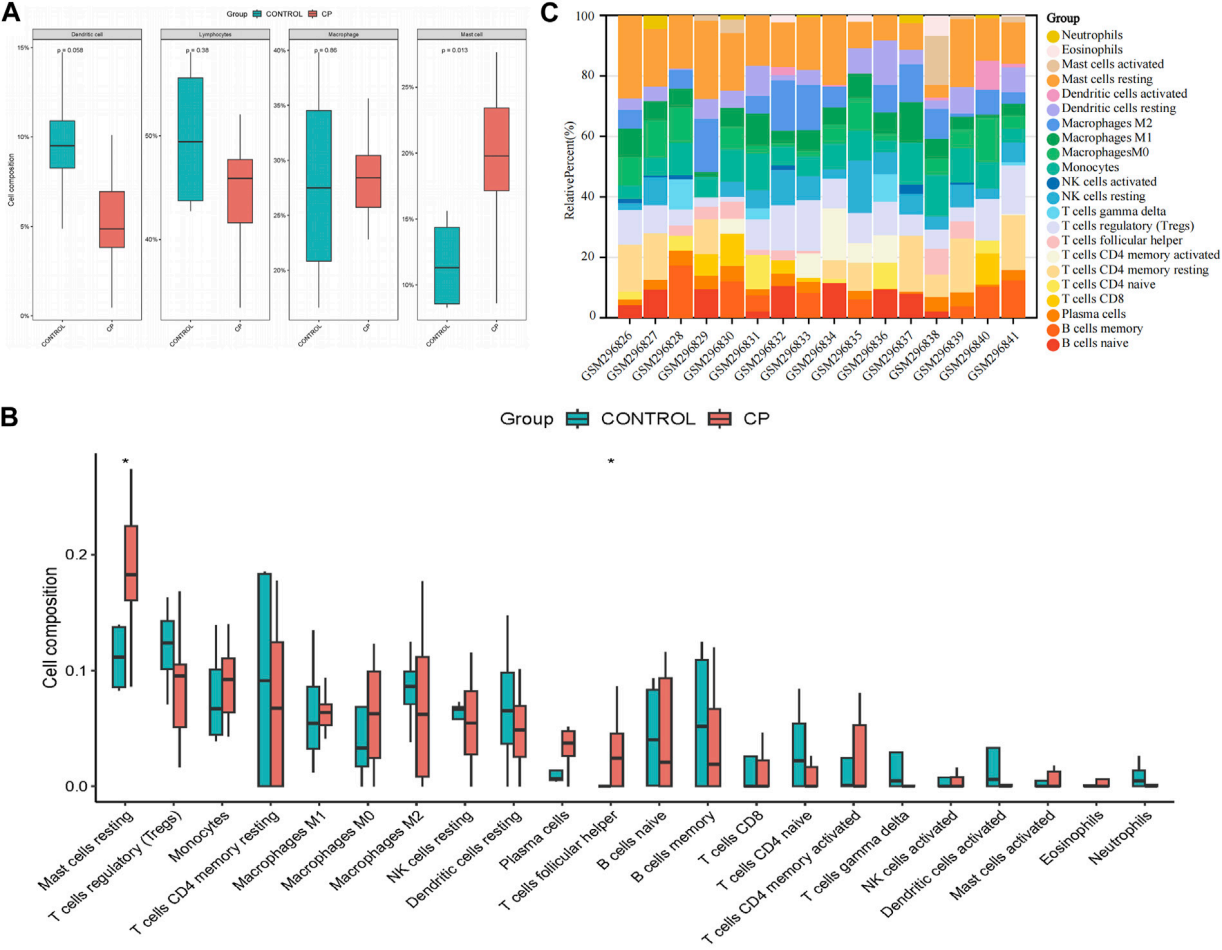
Evaluation and visualization of immune cell infiltration. **(A)** Boxplot of the proportion of four classes of immune cells; **(B)** Boxplot of the proportion of 22 types of immune cells; **(C)** Stacked bar graph of the proportion of 22 types of immune cells. NK, natural killer. *p < 0.05 compared with the controls.

The assessment of immune infiltration within the sample was conducted using robust bioinformatics methodologies, specifically the CIBERSORT algorithms. Compared to normal samples, samples from patients with CP generally exhibited a higher proportion of mast cells (*p* = 0.013), while Dendritic cells were relatively lower (*p* = 0.058, Figure 3A). In particular, CP patient samples often had a higher proportion of resting mast cells and T cells follicular helper (*p* < 0.05), suggesting a potential regulatory role in the immune response (Figures 3B, C). These findings highlight the complex interplay of various immune cell subsets and emphasize the importance of their interactions in shaping the immune landscape of the analyzed sample.

### Identification of co-expression gene modules in CP

**FIGURE 4:**
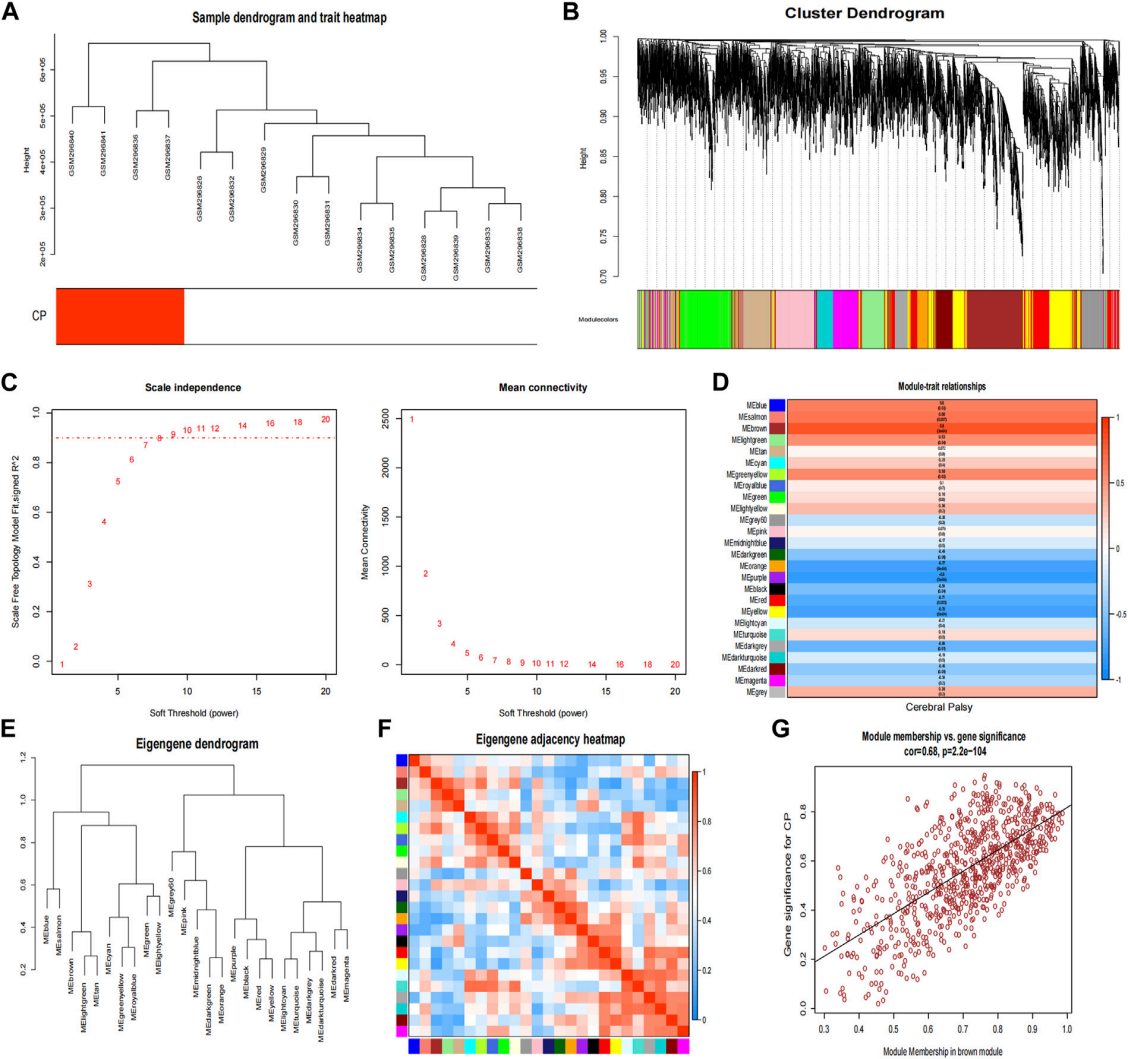
Weighted co-expression network related datasets construction. **(A)** Sample dendrogram and trait heatmap; **(B)** Gene dendrograms obtained by average linkage hierarchical clustering; **(C)** Analysis of network topology for various soft thresholds (β); **(D)** Module-trait relationships; **(E)** Clustering dendrogram of genes; **(F)** Module eigengene adjacency heatmap; **(G)** The correlation between the module membership (MM) and gene significance (GS) of the disease group of all genes in the brown module. The correlation value represents the absolute correlation coefficient between GS and MM. CP, cerebral palsy.

In the CP datasets, after excluding any outliers, we used WGCNA to identify co-expression gene modules among multiple genes (Figures 4A, B). To ensure that the network resembled a scale-free network, we calculated the soft-thresholding power, which was found to be 8 based on a scale independence of >0.9 (Figure 4C). By employing hierarchical clustering analysis and dynamic branch cut methods on the gene dendrograms, we grouped the genes into 26 modules (Figure 4F). The clustering dendrogram of the genes is shown in Figure 4E, where genes with similar characteristics are clustered together and represented by the same module color. Importantly, these modules were found to be independent of one another. Figure 4D provides a summary of the significance of all genes in each module with respect to CP. Notably, the brown module exhibited a significant association with CP and was selected for further analysis (*p* = 3e-04). The scatter plot in Figure 4G illustrated the relationship between CP gene significance and module membership, with a total of 762 genes being significantly associated with CP.

### Extract hub genes from DEGs and the hub module in WGCNA

**FIGURE 5:**
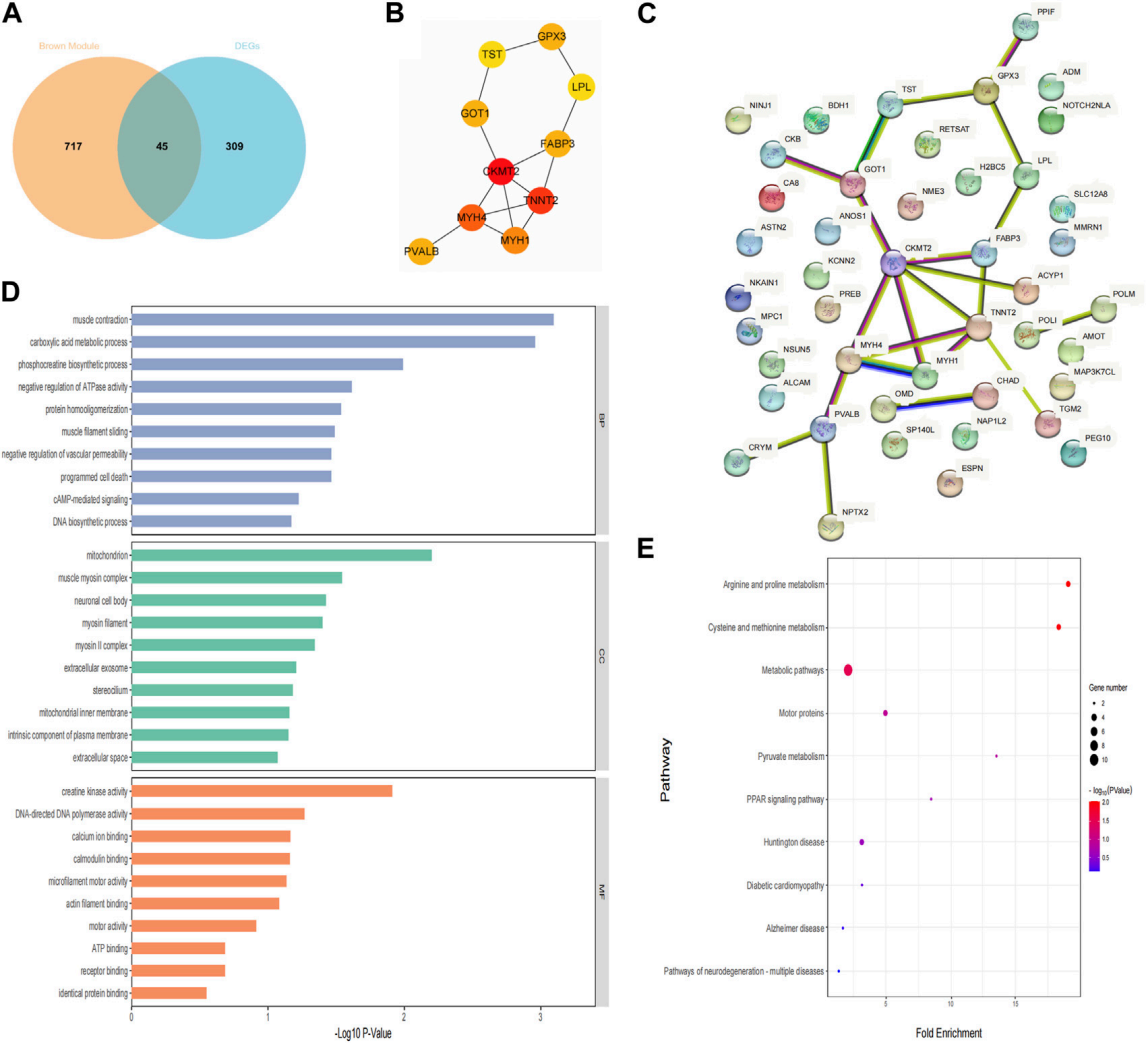
Hub genes identification and functional enrichment analysis. **(A)** The overlap of DEGs and module genes was shown as a Venn diagram; **(B)** The top 10 hub genes with the most correlations identified using CytoHubba; **(C)** PPI network of 45 hub genes generated by the Cytoscape software; **(D)** Gene ontology enrichment analysis; **(E)** The Kyoto Encyclopedia of Genes and Genomes pathway enrichment analysis.

Forty-five candidate genes were identified from the intersection of a venn diagram between two sets of the DEGs and WGCNA brown module (Figure 5A). To explore the biological features and significance of these 45 hub genes, GO and KEGG pathway enrichment analyses were performed (Figures 5D, E). The results of the analysis revealed that these hub genes were significantly related to various biological processes such as muscle contraction, carboxylic acid metabolic process, and phosphocreatine biosynthetic process. In terms of molecular function, the hub genes are associated with creatine kinase activity, DNA-directed DNA polymerase activity, and calcium ion binding. The enrichment analysis of cell component showed a focus on mitochondrion, muscle myosin complex, and neuronal cell body. Additionally, the KEGG pathway analysis indicated that arginine and proline metabolism, cysteine and methionine metabolism, and metabolic pathways were significant pathways in these 45 hub genes. These findings suggest that these genes are significantly enriched in energy metabolism-related pathways, indicating their potential role in muscular movement. For further analysis, a PPI network was constructed among the 45 candidate genes using Cytoscape software (Figure 5C). The MCC method in the CytoHubba plug-in was used to identify potential key genes. The top 10 Hubba nodes were collected for subsequent analysis (Figure 5B). Among the 45 genes, CKMT2, TNNT2, MYH4, MYH1, FABP3, PVALB, GOT1, GPX3, TST, and LPL were identified as the hub genes by the CytoHubba plug-in.

### Screening and validation of diagnostic markers

**FIGURE 6:**
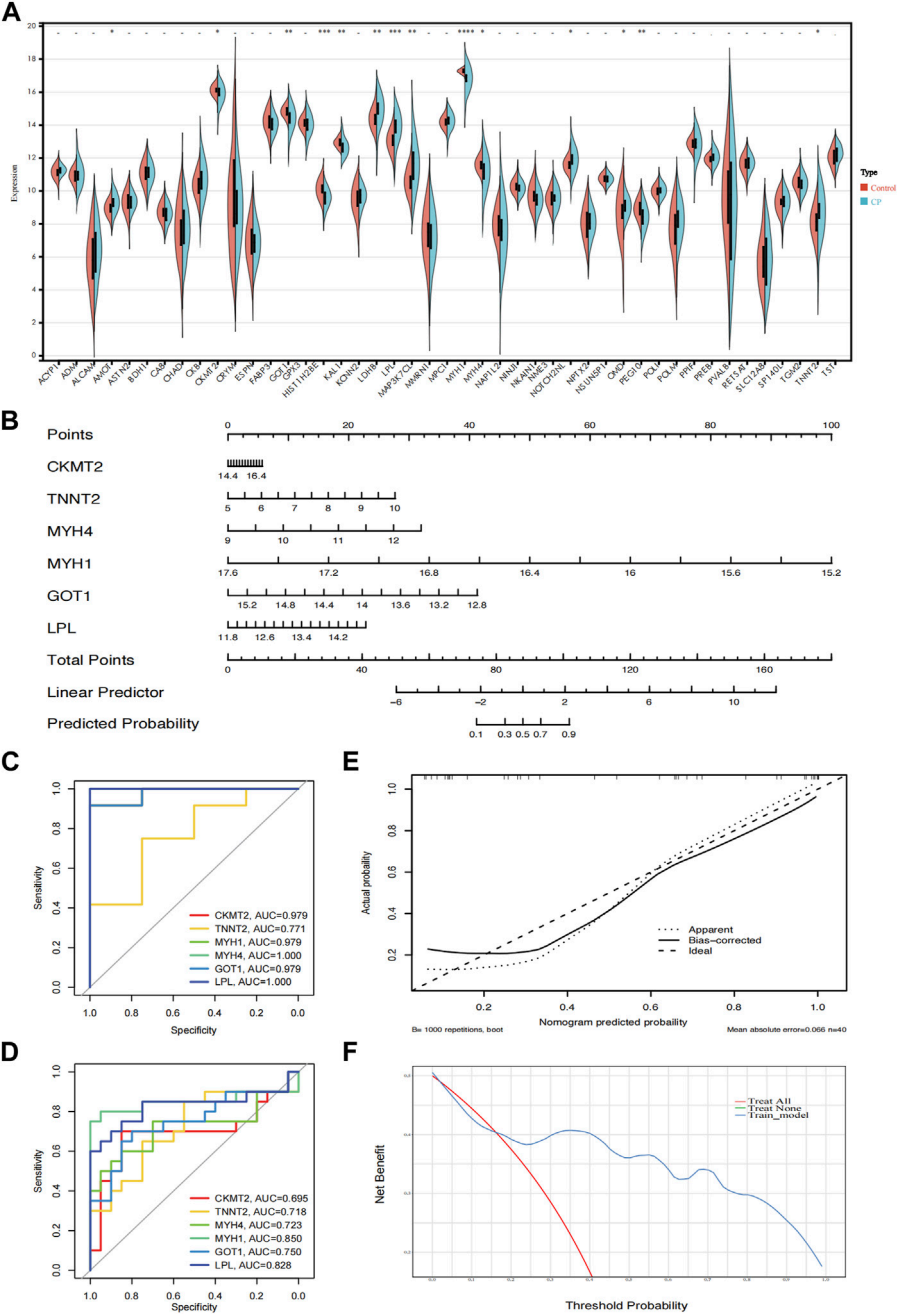
Validation of the hub genes. **(A)** The expression of 45 genes in the validation cohort (GSE31243); **(B)** A nomogram estimated CP risk in the training cohort by summing scores from each risk factor and positioning the total on the corresponding bottom line to calculate the probability of CP; **(C)** ROC curves of the training cohort; **(D)** ROC curves of the validation cohort; **(E)** The calibration curve shows the nomogram-predicted CP probability (x-axis) versus actual CP probability (y-axis). The diagonal dotted line represents perfect predictions, while solid lines represent nomogram performance. The closer the solid lines are to the diagonal, the better the prediction accuracy; **(F)** Decision curve analysis shows the prediction model’s net benefit (y-axis) against the threshold probability (x-axis), where the harm of false positives exceeds that of false negatives. Higher net benefit at the same probability indicates better clinical usefulness. CP, cerebral palsy. ****: *p* < 0.0001, ***: *p* < 0.001, **: *p* < 0.01, *: *p* < 0.05.

To further demonstrate the significance of the key genes in the module of interest, we assessed the expression of 45 candidate genes using muscle samples from the GSE31243 dataset (Figure 6A). Comparative analysis between the two samples revealed that 14 genes exhibited statistically significant differences in the CP sample. When the top 10 hub genes identified by MCC were analyzed together, we found 6 genes that were statistically different: CKMT2, TNNT2, MYH4, MYH1, GOT1, and LPL. This suggests that these six genes are important in relation to CP. Consequently, we developed a prediction model for CP in the validation cohort based on the expression of these six genes. The final model we obtained was as follows: prediction model = 104.2864 + 0.3745*CKMT2 + 0.8794*TNNT2 + 1.4529*MYH4 − 6.6211*MYH1 − 2.5241*GOT1 + 1.2096*LPL. Additionally, we created a nomogram to visualize the model and used a calibration curve to assess its accuracy. The nomogram is presented in Figure 6B, and the calibration curve is shown in Figure 6E (Mean absolute error = 0.066). The calibration curve of the nomogram for predicting CP risk demonstrated good agreement. Furthermore, the Hosmer-Lemeshow test, which evaluated the model, yielded a Chi-square value of 12.045 (*p* = 0.1492 > 0.05), indicating that the predictive model performed well. In addition, we compared the predictive value of the model with that of the six individual genes. The ROC curves revealed that the combined six-gene prediction had a higher value than the prediction based on a single gene (AUC = 0.905 in the validation cohort) (Figures 6C, D). Finally, according to the results of the decision curve analysis (DCA), the nomogram model provided a superior clinical benefit (Figure 6F).

### Potential drugs targeting the diagnostic genes

**FIGURE 7:**
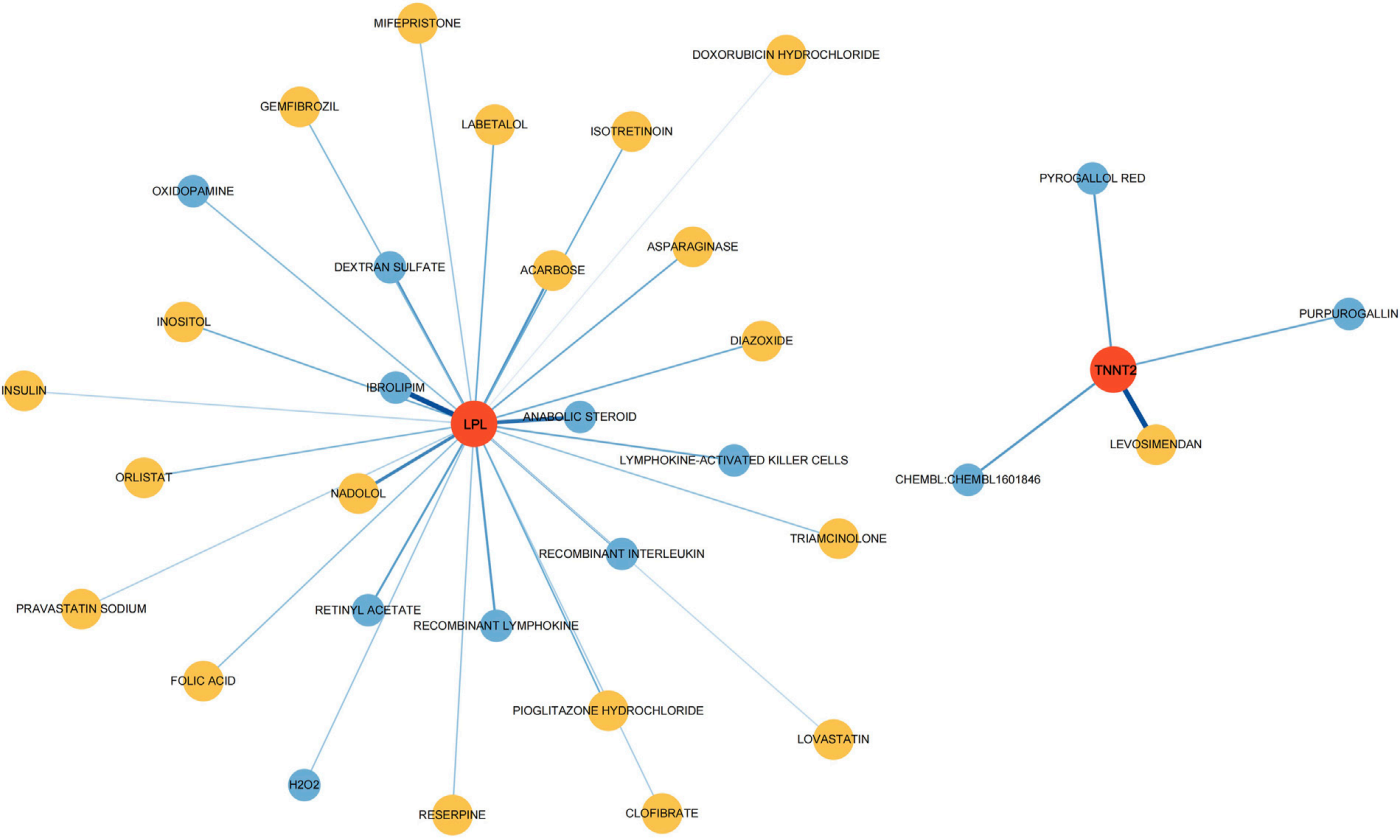
Drugs–hub genes interaction network. The red nodes represent genes, yellow nodes represent approved drugs, and blue nodes represent drugs not yet approved.

To investigate potential drugs for CP therapy, we conducted a search in the DGIdb database for drugs targeting the biomarkers. Our analysis revealed that 28 drugs targeting LPL and 4 drugs targeting TNNT2 were identified. Subsequently, we generated a gene-drug network consisting of 34 nodes, which is presented in Figure 7. Notably, regulatory approval has been granted to 19 drugs targeting LPL and 1 drug targeting TNNT2.

## Discussion

This study emphasizes the urgent need for early and accurate identification of biomarkers for CP to enhance diagnostic precision and improve patient outcomes. Through rigorous analysis, several potential biomarkers were identified, providing insights into the pathophysiological mechanisms related to CP. Developing predictive models based on these biomarkers offers opportunities for early diagnosis and personalized therapeutic interventions for CP. In our research, we identified 45 potential key genes through differential expression and WGCNA. Subsequent GO and KEGG analyses revealed that these genes are primarily involved in energy metabolism-related pathways in the development of CP, underscoring their crucial role in muscle movement. Further dataset validation identified CKMT2 as the key gene most closely associated with CP. Additionally, we established a predictive model for CP by combining five other significantly differentially expressed genes (TNNT2, MYH4, MYH1, GOT1, and LPL).

Previous studies had also identified differential genes and pathways associated with CP, which share many similarities with our research. Our study, along with those by Pingel and Robinson et al., identified genes related to energy production and muscle function as significant in CP [15, 16]. Genes involved in ECM structure and turnover have been emphasized in multiple studies. Increased ECM turnover and net collagen synthesis enable ECM remodeling as an adaptive response to the increased mechanical load and functional demands caused by spasticity [17]. Previous research had shown significantly lower LPL expression and increased intramuscular fat levels in CP patients, which was consistent with our findings [18, 19]. Additionally, Pingel et al.’s study highlighted the importance of calcium homeostasis in skeletal muscle movement and plasticity, finding distorted calcium ion handling in CP [11]. Stress, cell death, and autophagy also had contributed to the pathology of CP. Each study provided unique insights into the specific genes and mechanisms involved in CP pathology, underscoring the importance of genes related to energy metabolism, muscle function, and ECM structure in CP.

In our diagnostic model, CKMT2 was identified as the key gene most closely related to CP. The CKMT2 gene encodes mitochondrial creatine kinase, an enzyme crucial for energy metabolism in tissues with high and fluctuating energy demands, such as the brain and muscles. CKMT2 plays a primary role in maintaining cellular energy homeostasis by facilitating the reversible transfer of phosphate groups between adenosine triphosphate (ATP) and creatine [20]. This process allows for the storage and transportation of energy within cells, particularly in mitochondria-rich tissues. Additionally, mitochondrial creatine kinase is believed to be essential for maintaining mitochondrial morphology by stabilizing contact sites between the inner and outer mitochondrial membranes. Impaired activity of CKMT2 has been associated with the loss of mitochondrial membrane potential and apoptosis [21]. In the intact rabbit heart, a rapid and irreversible loss of CKMT2 was observed, which was directly related to the duration of ischemia. This loss of CKMT2 correlated with contractile dysfunction during reperfusion [22].

Further studies have demonstrated that CKMT2 overexpression protects against cellular oxidative stress damage, likely due to increased creatine kinase activity and its role in promoting mitochondrial integrity [23, 24]. CKMT2 is crucial for regulating energy production and utilization in the brain, ensuring a constant energy supply essential for neuronal function, neurotransmission, and brain health. Beyond energy provision, CKMT2 maintains cellular energy reserves and buffers against energy fluctuations. Variations or mutations in the CKMT2 gene may contribute to mitochondrial dysfunction, disrupting energy balance in neurons and potentially influencing the onset or severity of CP. Therefore, studying the correlation between CKMT2 variants and CP clinical features (such as severity, motor impairment patterns, or associated comorbidities) can deepen the understanding of disease subtypes and their pathological mechanisms, providing opportunities for personalized treatment. Exploring pathways aimed at regulating mitochondrial function or enhancing energy metabolism may serve as therapeutic strategies to alleviate symptoms or prevent the progression of related damage. Further large-scale genetic studies, functional analyses, and investigations into mitochondrial function will be essential to determine their significance in disease development and identify potential therapeutic targets.

The remaining five genes in the diagnostic model (TNNT2, MYH4, MYH1, GOT1, and LPL) also contribute to the pathology of CP through different mechanisms. TNNT2 encodes a component of the troponin complex critical for muscle contraction regulation, with variants linked to neuromuscular disorders [25]. In CP, TNNT2 variations might affect muscle tone regulation, contributing to motor impairments. MYH4 and MYH1 encode myosin heavy chain proteins, which are essential for muscle contraction. Alterations in these genes may impact muscle fiber composition or contractile properties, potentially leading to abnormalities in motor function and muscle tone observed in CP [26, 27]. GOT1 (Glutamic-Oxaloacetic Transaminase 1) is involved in amino acid metabolism [28]. Although its direct role in CP is not yet clear, disruptions in amino acid metabolism pathways could potentially affect brain development or neural function, thus contributing to the complex etiology of CP. LPL (Lipoprotein Lipase) plays a crucial role in lipid metabolism, affecting neurodevelopment and neuronal health [29]. Dysregulation of LPL may lead to changes in lipid metabolism, which correlates with the previously observed increase in intramuscular fat levels [18]. In conclusion, while the roles of TNNT2, MYH4, MYH1, GOT1, and LPL genes in CP are still under investigation, their involvement in muscle function, metabolic pathways, and potentially neurodevelopmental processes could contribute to the diverse clinical manifestations observed in individuals with CP. Variations in TNNT2, MYH4, and MYH1 may affect muscle structure, contractility, or neuromuscular junction function, contributing to motor impairments and muscle tone abnormalities. Meanwhile, genes such as GOT1 and LPL, involved in amino acid and lipid metabolism respectively, may indirectly affect neurodevelopmental processes and lipogenesis in muscle. Dysregulation of these pathways could impact substance synthesis and neuronal health within muscle, potentially contributing to the multifactorial nature of CP. Further research is needed to validate the differential expression of these genes and their direct impact on the pathogenesis of CP. Experimental models and functional assays are necessary to elucidate their specific contributions to neuronal development or muscle function. Additionally, studying the differential expression of genes and their potential association with birth complications may provide valuable insights into the etiology of CP.

Through the DGIdb database, we obtained potential therapeutic agents targeting the biomarkers. Purpurogallin (PPG) possesses significant antioxidant properties. By inhibiting the TLR4/NF-κB pathway and thereby attenuating endoplasmic reticulum stress and neuroinflammation, PPG demonstrates potential neuroprotective effects against cerebral ischemia-reperfusion injury [30]. Insulin, beyond its role in glucose metabolism, has shown neuroprotective effects and might influence brain development and neuroplasticity, which could be relevant in CP management. Lymphokine-activated killer (LAK) Cells and recombinant lymphokine have cytotoxic activity against tumor cells when activated *in vitro*, but their effects on CP remain unexplored. Levosimendan has a vasodilatory effect, and its potential impact on cerebral circulation and muscle tissue blood supply in CP patients needs further clarification [31]. Statins (Lovastatin, Pravastatin) have shown neuroprotective and anti-inflammatory effects, potentially beneficial in managing neuroinflammation in CP. Diazoxide, a vasodilator and potassium channel opener, does not have well-documented effects on CP but might influence blood flow or neural excitability. Triamcinolone, a corticosteroid, has the potential to suppress inflammation and immune responses, making it a potential option for managing inflammation-related aspects of CP. In a frozen shoulder rat model, the injection of triamcinolone acetonide has shown effective anti-fibrosis, anti-angiogenesis, and anti-inflammatory properties [32]. While these drugs show promise in affecting neurological functions or mechanisms related to CP, their specific impacts on CP patients require extensive clinical studies. Considerations such as dosage, duration, individual variability, and underlying pathology are crucial when evaluating their effects. Some drugs’ impacts on CP may not be well-documented or explored in clinical trials specifically for this condition, necessitating targeted research or clinical trials to evaluate their efficacy and safety in this population.

The study used samples from various muscle groups, with tissue collection sites as potential confounders. Different muscle groups exhibited unique gene expression profiles due to their physiological functions and fiber types [33]. Wrist muscles, crucial for fine motor skills and complex hand movements, showed a high gene expression in pathways involved in neuromuscular junctions, muscle contraction, and calcium handling [34, 35]. Conversely, hamstring and quadriceps muscles, involved in gross motor functions, exhibited increased gene expression in ECM tissue and muscle fiber composition for structural integrity and weight-bearing [36]. Additionally, elevated expression related to oxidative phosphorylation, muscle repair, and regeneration supported endurance and adaptive recovery [37, 38]. These differences highlight the unique needs of each muscle group and suggest personalized strategies for treating related diseases. However, obtaining muscle biopsy tissue from high-risk CP patients is an unavoidable challenge. Ethical considerations and strict informed consent procedures, especially for children, must be given primary consideration. The invasiveness of the surgery, along with the risks of postoperative infection, bleeding, and discomfort, may deter participation. Additionally, the medical fragility and anesthesia risks in CP patients complicate the procedure. Despite these challenges, muscle biopsies are crucial for studying the pathophysiology of CP and subsequently developing targeted therapies to improve muscle function and quality of life. Careful planning and ethical oversight are required, balancing the need for high-quality data with alternative, less invasive methods.

The CP prediction model based on hub genes demonstrates superior predictive power and accuracy compared to utilizing single genes. However, certain limitations related to the data must be acknowledged. The limited size of the cohorts in our dataset was a significant constraint, restricting the statistical power and robustness of our findings. Additionally, differences in age and sex between the control and CP groups represented potential confounding factors. Age-related gene expression differences and sex-specific biological variations can impact results, making it challenging to attribute observed differences solely to CP. Secondly, variability in the severity of CP may exhibit different molecular characteristics. Stratifying CP patients based on detailed clinical data and severity could help elucidate the relationship between CP severity and biomarker expression. It is essential to dynamically monitor changes in gene expression profiles throughout disease progression in longitudinal cohorts. Furthermore, variability among different muscle samples needs further clarification. In CP patients, muscle tissue often exhibits unique pathological changes such as increased ECM, fat infiltration, and heightened inflammation, which can affect gene expression outcomes due to differences in tissue composition. Isolating specific cell types or using single-cell RNA sequencing can provide a more precise understanding of the molecular basis of CP.

Our study highlights the importance of considering demographic variables, repeated measures, and tissue composition in biomarker research. Despite the limitations, our findings provide valuable insights into the molecular underpinnings of CP. Future research should focus on using larger, well-matched cohorts and advanced analytical techniques to improve the accuracy and applicability of biomarker discoveries. By addressing these factors, we can enhance the diagnostic and therapeutic potential of CP biomarkers.

## Conclusion

This study provides new insights into identifying potential biomarkers for CP and developing predictive models for early diagnosis and personalized treatment. Using comprehensive bioinformatics approaches, promising biomarkers (CKMT2, TNNT2, MYH4, MYH1, GOT1, and LPL) were identified, and robust predictive models for muscle sample markers specific to CP were developed. The findings highlight the importance of incorporating biomarker-based diagnostics into clinical practice to enable early and accurate diagnosis, leading to timely interventions and improved long-term outcomes. Future research should validate these biomarkers and models in larger cohorts and translate them into practical diagnostic tools and treatment protocols, ultimately enhancing the quality of life for individuals with CP and their families.

## Data Availability

Publicly available datasets were analyzed in this study. This data can be found here: https://doi.org/10.5281/zenodo.12599229.

## Footnotes

1
http://www.gsea-msigdb.org/gsea/downloads.jsp

2
http://david-d.ncifcrf.gov/

3
https://string-db.org/

4
http://www.cytoscape.org/

5
https://www.dgidb.org/
